# Danmu subtitling as a self-regulative practice: a descriptive discourse analysis of Bilibili danmu subtitles from ethical perspectives

**DOI:** 10.3389/fpsyg.2025.1577260

**Published:** 2025-07-30

**Authors:** Duo Zeng

**Affiliations:** School of Foreign Studies, Nankai University, Tianjin, China

**Keywords:** danmu subtitling, translation ethics, ethical conflict, online collaborative translation, descriptive discourse analysis, Bilibili

## Abstract

Translation ethics is an important aspect of Translation Studies that has gained increasing attention in recent years. From an ethical perspective, this study investigates the onscreen commenting system: danmu subtitling, an emergent non-professional and collaborative translation practice popular in Asian communities. Adopting, modifying and expanding Andrew Chesterman’s models of translation ethics, i.e., representation ethics, service ethics, communication ethics, norm-based ethics and ethics of professional commitment and proposing an ethics of digital technology, this study delves into how ethical principles explain the user-generated danmu subtitles. Descriptive discourse analysis is performed in this study observing an episode of *Tamara’s World* (uploaded with no subtitles or translations), an English variety show, examining 296 translation-related instances among 1,490 danmu comments. Findings indicate that multiple ethical models elucidate danmu subtitling practices, which encounter distinct ethical conflicts. Consequently, adaptive regulation is proposed to resolve these conflicts.

## 1 Introduction

In the contemporary media landscape, danmu, an emergent commentary system featuring “user-made comments... scroll across the screen like an animated subtitle-track” (Johnson, 2013, p. 297), facilitates participatory viewing and mass writing. In terms of audiovisual communication, official and commercial channels may delay or overlook content that is time-sensitive, topic-specific, or relevant to minority groups. Therefore, grassroots forces utilize danmu subtitling to address the demand for media accessibility in specific contexts, such as assisting individuals who lack proficiency in a second language and facilitating intercultural communication.

In Translation Studies, scholarly attention to danmu subtitling is gaining ground. While semiotics has significantly contributed to existing research (Pérez-González, 2019; Yang, 2020, 2023), various aspects of danmu have been explored. These include audience reception studies through danmu analysis (Lu and Chen, 2024), empirical case studies of multimodal and interactive danmu subtitling (Wu and Fitzgerald, 2023), and the role of danmu as paratext (Yang, 2023). Nevertheless, despite recognition of its inherent ethical implications, a gap exists: few studies systematically examine danmu subtitling through the lens of translation ethics frameworks. Addressing this gap is essential, as danmu subtitling represents a pervasive and influential form of collaborative translation in digital media, demanding rigorous ethical examination.

Translation ethics has attracted sustained scholarly attention since its inception over three decades ago (Berman, 1984). Accelerated globalization and technological innovation have intensified ethical challenges in translation practices. Existing scholarship explores this domain extensively, spanning foundational ethical frameworks, context-specific ethical issues, agentic and institutional ethical considerations, ethical reflection on education, and emergent ethical challenges precipitated by technological mediation (Bowker, 2020; Desjardins and Florentin, 2024; Kenny, 2011; Koskinen and Pokorn, 2021).

Scholarship interrogates ethical issues in specific contexts and platforms, with research focusing on ethics of fansubs (Díaz- Cintas and Muñoz Sánchez, 2006), ethics of crowdsourcing (Dolmaya, 2011), interpreting ethics in fragile environments (Gallai, 2019), ethical codes of community translation (Drugan, 2011), ethical cooperation in Wikipedia (Hu, 2024), and ethical insights from Yeeyan Gutenberg Project (Pan and Xiao, 2024). Nevertheless, despite the existence of fragmented ethical discussion (e.g., Díaz-Cintas, 2018), scholarly investigation of danmu subtitling through an ethical perspective remains underdeveloped. This gap is particularly salient given danmu subtitling’s unique characteristics— (pseudo) real-time, collaborative, anonymous, and embedded within the viewing experience—which generate distinct ethical features and challenges not fully captured by existing frameworks.

Danmu subtitling, as a form of ethical behavior, introduces significant ethical considerations into the media landscape. Ethical decision-making plays a crucial role in translation, particularly in complex and challenging contexts (Drugan, 2011). In the context of danmu subtitling, these ethical considerations become even more pronounced, as viewers are no longer passive recipients of content. However, they are at the same time producers empowered by the danmu interface to actively engage with the video and interactions. This necessitates a heightened awareness of ethical considerations. For instance, the content of danmu should avoid defamation or harassment, and maintain a civil tone that does not disrupt the viewing experience for others. The anonymity provided by the platform and lack of strict societal oversight may empower some danmu subtitlers to act casually, leaving more of their decision influenced by individual ethical self-regulation in distinguishing between right and wrong.

Given this context, the present study makes a significant contribution by systematically applying, adapting, and extending Chesterman’s (2001) models of translation ethics—representation, service, communication, norm-based, and professional commitment—to the novel phenomenon of danmu subtitling. Crucially, it also proposes and tests an “ethics of digital technology” to account for the affordances and constraints inherent in the danmu interface. This theoretical innovation allows us to rigorously investigate how ethical models manifest in and explain the emergent, collaborative translation process undertaken by danmu subtitlers.

By pioneering this ethical analysis, the study provides crucial theoretical and empirical insights that significantly enrich both Media Studies and Translation Studies. It advances our understanding of participatory media culture, collaborative translation ethics in digital spaces, and the complex interplay between technology, user agency, and ethical decision-making in emergent translation practices.

## 2 Danmu subtitling as an emerging mode of online collaborative translation

“Danmu” (“弹幕” in Chinese) is a video commenting system that originated in 2007 by Niconico, a Japanese video-sharing website. This system allows viewers to send their comments in real-time, which are then displayed as floating (primarily) or fixed subtitles over the video content. Since its inception, it has evolved and gained significant popularity in Asian communities. Although the system is no longer new, it has only in recent years become a subject of interest due to its unique features and the interactive viewing experiences it facilitates.

Various platforms and their technological affordances have facilitated diverse levels of viewer engagement with video content (Lange, 2007, p. 361). Among these, the danmu interface is particularly notable for promoting viewer participation in the construction and negotiation of video content, thereby enhancing engagement and interaction through its multimodal semiotic affordances (Yang, 2021).

By leveraging the affordances of the danmu interface, danmu comments—which represent the manifestation of this system—have given rise to a distinctive aspect of video consumption known as pseudo-synchronicity. This phenomenon, described as “a feeling of ‘live’ viewing via a sense of virtual time shared between users.” (Johnson, 2013, p. 297), creates an immersive experience that resembles synchronous communication despite being asynchronous.

Though research on danmu is growing to be prolific and interdisciplinary, many aspects of danmu have remained unknown. By defining danmu comments as a multifaceted form of user-generated content that enhances the viewing experience by providing translations, emotional reactions, critical analysis, informational insights, and social interaction (hereinafter referred to as danmu comments), and danmu subtitles as the danmu subtitlers’ collaborative production of projecting translation-related danmu comments (hereinafter referred to as danmu subtitles), this study aims to contribute to the growing body of knowledge surrounding danmu culture and its potential to inform translation practices and theories and media studies.

As a derived activity of danmu, danmu subtitling is still an emerging field, and research on it is limited in scope and volume. Early but not detailed mention of the danmu subtitling can be found in Wu et al.’s (2018) comparison of danmu comments and traditional forum comments, in which the group of danmu subtitlers, who unofficially translate foreign languages into Chinese utilizing danmu, is identified as one of the major active users. Since then, the field has enhanced its scope overtime on its way to a fully-fledged research area.

Social semiotic perspective plays a pivotal role in guiding the exploration of meaning-making ensemble enabled by danmu subtitling. Pérez-González (2019) notices the emancipation of subtitling from media industries thanks to the advent of digitization. In his study, Pérez-González (2019) examines danmu as the interplay between semiotic technology (design) and social practices (rhetoric). He emphasizes the shift from the traditional “representational” role of danmu subtitles to an “interventionist” role, which reconfigures multimodal discourses (Pérez-González, 2019, p. 111).

Descriptive case studies and multimodal discourse analysis are two commonly used methods in danmu subtitling research. Yang’s (2021, 2022) multimodal discourse analyses of danmu-enabled new grassroots translation practice further investigate this semiotic and social practices’ meaning-making process by fine-grained, data-led, and typical-themed case studies. Wu and Fitzgerald (2023) conducted a thorough investigation into user interaction while viewing danmu subtitles on top of officially untranslated videos, employing digital conversation and multimodal discourse analysis.

Recent advancements in the analysis of danmu subtitling have begun to explore its multifaceted nature through varied theoretical frameworks and interdisciplinary perspectives. For instance, Yiheng Zhao’s semiotic theory, characterized by its focus on the dynamic and interpretative potential of signs, and his category of co-texts are imported to approach the danmu-mediated online video consumption (Yang, 2023). Chen (2023) introduces the concept of “danmu-assisted learning through back translation,” viewing danmu subtitling as a tool serving specific purposes. Qiao’s (2024) study delves into the intricate relationship between macro and micro contexts, particularly within the context of Chinese fans’ engagement with danmu subtitled videos featuring Cardi B. Of particular interest in this study is the observation of danmu comments, which function as a bridge between the macro and micro contexts in cyberspace.

## 3 Danmu subtitling considered as an ethical behavior

Ethics, from its inception, can be defined as the systematic examination of moral principles and values (Koskinen and Pokorn, 2021, p. 2). In the realm of Translation Studies, ethics specifically refers to the branch that seeks to understand the ethical dimensions of translation practices, including what constitutes good and bad, and right and wrong translations. According to Chesterman (2018, p. 443), “translation ethics” (or “translator ethics”) refers to the set of accepted principles according to which translation should follow and hence the norms governing what translations should be like.

According to Pym (2012, p. 134), the ethical responsibilities of translators can be framed within a broader context of cooperation. Pym (2000, p. 184) argues that translator ethics are more concerned with the interactions among translators, clients, editors, payment rates, immediate reception, distribution networks, and intercultural spaces—where professional and commercial elements intersect—than with distant authors and readers. In the case of danmu subtitling, these ethical considerations align closely with Pym’s framework, as it involves a collaborative translation practice that is volunteer undertaken by non-professional danmu subtitlers.

Technological innovation and democratization have brought about new translation technologies and emergent forms of translation, leading to the emergence of new ethical concerns. These include concerns about the decline in the professional status of translators (Dolmaya, 2011); the exploitative aspects, despite the apparent mutual benefits, of online collaborative translation (Zwischenberger, 2022); the ethical challenges arising from team interactions (Hu, 2024); and ethical considerations stemming from risk management (Pan and Xiao, 2024).

Danmu subtitling, as facilitated by the danmu interface, raises significant ethical considerations that extend beyond technological aspects to include questions of authorship, copyright, and power dynamics. For instance, the absence of entry criteria and the continuous enrollment of danmu subtitlers cast doubt on authorship, and it erodes professional subtitling and poses a threat to professional subtitlers. As the matters of resource ownership and power disparities associated with certain online collaborative translation practices (Hu, 2024), the copyright issues surrounding both the source materials and their translations remain a disputed topic. However, the imbalance of power relations is not as pronounced in danmu subtitling, given that danmu subtitlers primarily engage voluntarily and can terminate their work at any time.

This study approaches danmu subtitling from an ethical perspective, recognizing the crucial role of danmu subtitlers’ ethical decision-making in danmu subtitling. Drugan (2011) has observed that ethical decision-making is particularly significant in complex and challenging contexts, where different priorities are revealed in professional and non-professional codes of practice. Additionally, Chesterman (2018, p. 443) highlights that axiological issues, including personal and professional ethics, significantly influence decision-making processes. These insights provide a foundation for examining danmu subtitling, where danmu subtitlers must navigate between different ethical values.

## 4 Research design

Descriptive discourse analysis is a qualitative research method that examines how language constructs meaning and mediates social realities. Brown and Yule (1988) establish that this method systematically identifies distinctive linguistic features within specific text or discourse. This study employs descriptive discourse analysis to examine the ethical dimensions of danmu subtitling, as it allows for an in-depth exploration of the textual and contextual elements of danmu subtitling.

### 4.1 Theoretical framework: Chesterman’s models of translation ethics

Linking the concept of the norm to professional ethics and examining different models of translation ethics, Chesterman (2001) outlined four models founded on four concepts: truth (representation), loyalty (service), understanding (communication), and trust (norms), and put forth an additional model, ethics of professional commitment, a virtue supporting the striving for excellence. Considering the role of humans in the translation process is significantly influenced by digital technology in the modern age, this study proposed an ethics of digital technology.

The production of danmu subtitling, as user-generated content, involves complicated ethical decisions that a single translation ethics model may fail to capture its full. Also, danmu subtitling occurs on social media platforms, where the interaction between danmu subtitlers and other viewers is vibrant. This interaction can significantly impact the ethical decision-making of danmu subtitlers, making it necessary to consider different ethical models to explain their behavior.

Chesterman’s models provide a comprehensive framework for understanding the ethical considerations involved in danmu subtitling. However, applying these models to danmu subtitling, a form of user-generated content, requires addressing several mismatches between the original models and the specific context of danmu subtitling. The connotation and application of these models within danmu subtitling are demonstrated as follows:

#### 4.1.1 Ethics of representation

The ethics of representation are rooted in faithfully translating without making any changes. It emphasizes the value of allowing the original meaning to be preserved without domesticating it.

The faithful translation in this case is unlike the translation of sacred texts or EU translation services, as elucidated by Chesterman (2001). In accordance with Chesterman’s assertions (2001), an ethics of representation underscores the significance of fidelity and truth. Essentially, the translator is obliged to faithfully and genuinely represent the content of the source text, adhere to the intentions of the source author, and even embody the essence of the source culture, akin to a dependable mirror reflecting the original intent with precision.

Although fidelity is a pretheoretical notion and Translation Studies scholarship tends to favor other terms (equivalence, accuracy etc.), there is the enduring argument of adhering to ‘loyalty’ towards the author and maintaining ‘faithfulness’ to the intended meaning (Munday, 2016, p. 53). Various translation methods reflect differing levels of fidelity. For instance, in Newmark’s (1988) flattened V diagram, translation methods such as word-for-word translation, literal translation, faithful translation, and semantic translation all demonstrate an emphasis on the source language.

In the case of danmu subtitling, translation endeavors can be faithful renderings of the original video without domestication, employing translation methods such as word-for-word translation, literal translation, faithful translation, and semantic translation.

#### 4.1.2 Ethics of service

The ethics of service view translation as a commercial service and prioritizes loyalty to the client and also to target readers and the original writer. In contrast, danmu subtitling is often performed by volunteers motivated by community engagement rather than commercial interests. To address the mismatch, the ethics of service can be reinterpreted to emphasize that danmu subtitling constitutes a voluntary service.

Conventional translation ethos frequently prioritizes neutrality and non-involvement (Baker and Maier, 2014, p. 3), necessitating translators to adhere to their professional ethical standards. In contrast to contractual ethics, the ethics based on the concept of treating translation as a voluntary service prioritize loyalty not to the client, target reader, or original writer, but to personal considerations, such as the desire to contribute to a community, the pursuit of a creative hobby, or the fulfilment of personal goals.

For instance, the utilization of danmu subtitling can be interpreted as a discretionary service implicitly encouraged by video-sharing platforms, given its capacity to broaden the audience reach of videos and enhance economic profits, akin to the phenomenon of crowdsourcing translation observed on Facebook (Zwischenberger and Alfer, 2022). In this regard, the platforms are unable to enforce stringent standards or regulations and danmu subtitlers are primarily driven by a sense of “inner satisfaction” (Dam and Zethsen, 2010, p. 204), which includes contributing towards a collective benefit. However, as observed by Zwischenberger (2022), online collaborative translation possesses an exploitative nature, despite its apparent benefits and danmu subtitling confronts similar ethical dilemmas.

#### 4.1.3 Ethics of communication

The ethics of communication focus on effective communication with others. Translators who follow this approach prioritize their loyalty to intercultural communication rather than to the source text or target readers.

As the potential of social subtitling for individuals lacking language skills in the target second language (L2) (Talaván Zanón and Ávila Cabrera, 2016), the significant reflection of the communication ethics echoes the core features of danmu subtitling, i.e., facilitating communication across linguistic and cultural barriers.

For Pym (1997), intercultural communication aims to foster mutual benefits through collaboration, and the ethical aspiration of translation lies in enhancing such intercultural cooperation. Consequently, an ethical translator strives to translate in a manner that maximizes this collaborative effort. Additionally, Pym (1997) observes that an ethical translator may occasionally deem it more advantageous to refrain from translation altogether and instead recommend alternative modes of communication, such as acquiring proficiency in the other language (Chesterman, 2001, p. 141).

These observations justify the diverse translation methods employed by danmu subtitlers, as they align with the ethical aspiration of enhancing intercultural cooperation. For instance, danmu subtitlers may employ adaptation to ensure the content is understood in the target culture, or supplementation to fill the cultural gap. Each method serves a specific purpose, such as maintaining cultural authenticity or ensuring clarity and comprehension, thereby supporting the broader goal of intercultural communication.

#### 4.1.4 Norm-based ethics

Norms arise from cultural contexts, making it the primary lens through which translation expectation, production, and reception are evaluated. Norm-based ethics rely on accepted norms in a particular culture to determine what a good translation should look like. The norms define the characteristics of acceptable translation products, highlighting how they differ across various periods and cultures (Chesterman, 2001). It recognizes that translation is not merely a linguistic exercise, but a cultural exchange that requires sensitivity and respect for the norms of the target culture. In this regard, danmu subtitlers are expected to be cultural mediators, bridging the gap between source and target cultures.

While norms offer pragmatic frameworks for ethical decision-making, their formulation remains inextricably tied to power dynamics, consistent with the view that politics permeates all realms of life through “relations of conflict and power” (Squires, 2004, p. 119). Translation norms—whether institutionally imposed (e.g., EU standardization), market-driven (e.g., anglophone publishing dominance), state-regulated (e.g., licensed translator systems), industry-mandated (e.g., professional associations), or emerging organically through social practice—reflect power structures that influenced what is considered ethical. For instance, when norms align with dominant power (e.g., colonial-era translations suppressing Indigenous voices), seemingly “ethical” conduct actually perpetuates epistemic violence (Spivak, 1993).

#### 4.1.5 Ethics of professional commitment

The ethics of professional commitment emphasize the importance of practitioners being committed to the values of the translation practice. This commitment is a virtue that supports the pursuit of excellence and being a good translator.

Chesterman’s (1997: 172) deontic logic of translation ethics identifies values such as integrity, truth, clarity, understanding, and trust as guiding principles for ethical decision-making, while certain virtues influence the practitioner’s decision-making process. Essential virtues for ethical decision-making include a genuine desire to act rightly, alongside creativity, cultural sensitivity, and a sense of social responsibility. This entails striving not only for technical competence as a ‘good translator’ but also for responsible engagement with the text, its audience, and broader societal implications. Consequently, commitment can be viewed as a unifying force that binds practitioners to the values inherent in the translation profession (Chesterman, 2001, pp. 146–147), demanding a constant negotiation between fidelity to these values, the exercise of personal creativity, and the demands of social responsibility, especially in evolving contexts. For instance, behaviors observed in online contexts, such as danmu subtitlers engaging in self-correction, often stem from a desire for accuracy and quality (reflecting professional commitment). However, the dynamic, interactive nature of platforms like danmu also creates space where translators might navigate tensions between strict fidelity, creative expression, cultural adaptation, and audience engagement, highlighting the need for the balance discussed above.

#### 4.1.6 Ethics of digital technology

This framework addresses the integration of human expertise with technological tools in translation, such as examining the ethical implications of AI translation, machine translation (MT), and automatic subtitle generation. As is observed that with the development of digital technologies, there are ongoing ethical issues, such as whether human translators will be replaced by technology or collaborate with it, as well as concerns related to data safety and copyright. This framework critically examines how technologies challenge core translation ethics: e.g., whether MT undermines translators’ creative labor, whether algorithmic bias risks accuracy declines, and how automation impacts accountability for quality and cultural sensitivity. Translators must therefore leverage technology while upholding ethical standards—scrutinizing AI outputs for bias/errors and asserting creative agency where automation falters.

Supported by the danmu interface’s technological affordances, the danmu interface exemplifies these tensions: While its real-time interactivity fosters community engagement, the pressure for instantaneity may limit output quality, risking accuracy and constraining subtitler creativity. Furthermore, anonymity can encourage candid contributions but also reduce accountability—potentially enabling unvetted wrong translations or ethically ambiguous adaptations.

For example, a unique translation perspective is enabled by danmu subtitling, which can be described as an omniscient perspective. Unlike traditional translations, certain danmu subtitles may adopt a third-party perspective. Instead of directly translating the subjects’ words, subtitlers use terms like “they” or “the characters” to represent the speakers. This approach creates a sense of detachment and allows viewers to perceive the dialogue from a more objective standpoint. Additionally, danmu comments appear dynamically, flashing in and out on the screen, which may influence subtitlers to opt for more concise translations (e.g., gist translation rather than communicative translation).

Ethical conflicts inherent in these models necessitate recognition and resolution. Chesterman’s (2001) analysis identifies predominant models of translation ethics, each presenting distinct values and engendering significant conflicts. These conflicts arise from incompatible core values, differing scopes of application, and divergent philosophical foundations, revealing the limitations of each model.

As Moser (1988) demonstrated, ethical conflicts stem from incidents violating an individual’s personal sense of right and wrong, where personal values serve as crucial determinants. Within danmu subtitling, diverse practitioners contribute heterogeneous value systems. Consequently, individual subtitlers’ ethics may conflict with platform policies, video creators’ intentions, or other danmu subtitlers’ stances.

Such ethical conflicts can lead to adverse outcomes. Thorne’s (2010) research correlates ethical conflict with employee stress, lack of commitment, absenteeism, and turnover intention. Applied to danmu communities, unresolved conflicts could similarly contribute to issues such as stress and disengagement among subtitlers. Such conflicts also carry psychological consequences: Kammeyer-Mueller et al. (2012) demonstrate that institutional pressures contradicting personal ethics cause distress and exhaustion. In danmu subtitling contexts, pressures to engage with controversial content, adhere to potentially ambiguous community guidelines or manage conflicting audience expectations via danmu could similarly trigger such psychological burdens for those involved. Brown et al. (2022) establish that while ethical conflicts disrupt team cohesion, they may stimulate moral cognition and refine ethical decision-making. This suggests that ethical conflicts within danmu subtitling could disrupt collaboration, yet they may also serve as catalysts for deeper ethical reflection and improve decision-making processes.

The unique affordances of the danmu interface—such as real-time overlay, anonymity (often), high visibility, and potential for rapid interaction—create specific conditions where these general mechanisms of ethical conflict are activated. Addressing ethical conflicts in danmu subtitling therefore necessitates formulating resolutions informed by understanding their underlying mechanisms. This study proposes that effective resolutions should transition from static to adaptive resolutions. Such self-regulation must accommodate the specific contextual needs of danmu subtitlers. For instance, when Communication Ethics takes precedence, danmu subtitling operates simultaneously as a cultural bridge and an information dissemination tool. Consequently, subtitlers bear responsibility not only for conveying content but also for evaluating its social implications, including potential misunderstandings or exacerbation of cultural conflicts.

### 4.2 Research questions

This study foregrounds the practice of danmu subtitling as ethical behavior, necessitating explication from an ethical perspective. The research question is set out as follows:

- How and to what extent a model designed for professional translation be used to identify and explain danmu subtitling, a non-professional translation? Specifically, how can Chesterman’s translation ethics framework applied to the analysis of danmu subtitling and aid in explaining the decision-making of danmu subtitlers?
- What ethical conflicts occur in danmu subtitling? What practical resolutions can help address them?

### 4.3 Video sample

The data set for this case study includes a video labelled with the keyword “生肉” [raw meat], which refers to untranslated foreign language videos on the Bilibili platform. Being the world’s most engaged video-sharing platform, Bilibili has registered 96.5 million daily active users in the second quarter of 2023 (Bilibili, 2023).

Given the popularity of entertainment genre videos on video-sharing platforms and their need for translation, a search was conducted on Bilibili on August 24, 2023, using the keyword “raw meat” to identify untranslated foreign language videos within the entertainment category and sorted by default comprehensive settings. Search results were refined by excluding short clips, and focusing on videos longer than 60 min.

The video series “有钱人的世界” (Tamara’s World), a reality series, was chosen for its high ranking on Bilibili, with the most views and a relatively higher number of danmu comments. It is expected that a more popular video with higher viewer engagement will offer deeper insights into the underlying reasons for the behavior of danmu subtitlers, who act as ethical agents. This expectation is based on the premise that increased popularity and engagement typically correlate with a higher volume of danmu comments and a more diverse range of interactions among viewers.

As of August 24, 2023, the video had accumulated over 294,000 views and received 3,084 danmu comments. Episode 1 was selected from the six episodes because it accounted for nearly half of the series’ total danmu comments (1,490 comments, or 48.31%).

**Figure 1:**
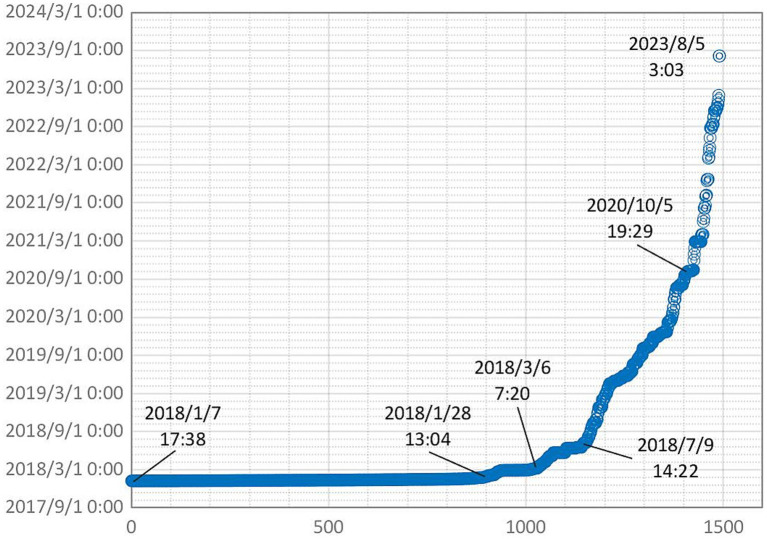
Time-based danmu comments distribution of *Tamara’s World* season 1 episode 1.

Figure 1 illustrates the rapid increase in danmu comments for the episode 1 video since its release on January 07, 2018, with the growth rate declining over 20 days.

### 4.4 Operational procedures

1 Downloading materials and retrieving data.

The free PC software Jijidown was utilized to download the video and its corresponding danmu file from Bilibili. Subsequently, the danmu file was imported into an Excel spreadsheet for data annotation and analysis.

2 Classifying danmu comments.

Drawing from Zhang and Cassany’s (2020, p. 491) taxonomy, danmu comments can be categorized into three broad groups: video, meta-danmu, and viewer-related danmu comments, and can be further classified regarding its specific topics in given contexts.

Two trained annotators independently classified danmu types using the predefined criteria. To minimize cognitive biases, a structured reconciliation protocol was implemented: annotation → cross-examination → discussion → agreement. All discrepancies underwent iterative discussion: annotators cross-examined contested cases and refined classifications through mutual debate until full agreement.

3 Applying, modifying, and extending Chesterman’s theoretical framework and analyzing.

Drawing upon Chesterman’s five models of translation ethics to the case study, the annotation criteria and examples are outlined below (Table 1).

**Table 1:** Annotation criteria and examples of translation ethics models.

Model of translation ethics	Definition in this study	Example
Ethics of representation	Faithful translation allows slight changes.	Original audio: I remember she looked beautiful Danmu subtitle 399: 我记得她很美 [I remember she was very beautiful]
Ethics of service	Translation as a voluntary service.	Danmu subtitle 28: 随便翻翻 大家随便看看 [Just a casual translation for everyone to take a look.]
Ethics of communication	Translation as an effective way of communicating with others.	Original audio: They say that the triangle is the strongest shape known to the mankind. So we are gonna to stick to three of us. Danmu 231: 我们三个组成了家, 大家都说三角形是最稳定的结构 [The three of us form a family, and everyone says that triangles are the most stable structure.]
Norm-based ethics	Priority is given to accepted norms in a particular culture, such as identifying the speaker’s name.	Original audio: Sophia: Just use your muscles. Tamara: I’m trying to use my muscles. Danmu subtitle 904: 菲:你应该用到你的肌肉 ta:我努力用肌肉的力量了 [Fei: You should use your muscles. ta: I tried hard to use my muscle strength]
Ethics of professional commitment	Emphasize the importance of practitioners being committed to the values of the translation practice.	Danmu subtitle 730: 翻译错了, 是sort of, 不是软软的 [The translation is wrong, it’s sort of, not soft.]
Ethics of digital technology	Highlights the integration of human expertise with technological tools, such as translation of omniscient perspectives or use of gist translation to accommodate the temporal nature of danmu interfaces.	Danmu subtitle 649: 他们为了女儿建了一个泳池就为了她能时而去玩[They built a swimming pool for their daughter so she could play there from time to time.]

It is noteworthy that a single danmu subtitle may reflect multiple translation ethics models, with priority given to the most prominent feature, and the same procedure of consensus-seeking employed. For instance, in Example 1, danmu subtitles 34 and 35 can be seen as illustrating norm-based translation ethics or ethics of communication. Both danmu comments are from the same user, who consistently uses English captions followed by Chinese translations across 18 danmu subtitles, resembling bilingual subtitles common in (Chinese) streaming media (Gouleti, 2023; Hsiao, 2014; Liao et al., 2020; Zhang, 2013). This consistent adherence to the bilingual format represents a normative ethical commitment: the subtitler prioritizes fulfilling an implicit duty to conform to established community (cultural) practices and expectations regarding accessibility and presentation, viewing this conformity itself as an ethical good. Despite this overarching norm, the specific Chinese translation in danmu subtitle 35 focuses on conveying the general meaning rather than a literal translation, as seen in the translation of “partying” as “嗨” [having fun], emphasizing the sense of having fun over the exact term “派对” [partying]. Thus, while operating within a norm-based ethical framework demanding bilingual presentation, the subtitler simultaneously exercises ethical judgment aligned with communication ethics, prioritizing functional equivalence for the target audience. This example’s annotation prioritizes the most prominent feature, namely norm-based translation ethics, understood as an ethical obligation to follow prevailing conventions.

**Example 1**

Danmu subtitle 34 I’ve had a lifetime of partying

Danmu subtitle 35感觉已经嗨了一辈子了

## 5 Results and discussion

In the subsequent section, a comprehensive analysis will be conducted, encompassing a general overview, a detailed examination of various ethical models applied in the explanation of danmu subtitling, and a discussion of ethical conflict emergence and resolution.

### 5.1 General analysis

Tables 2, 3 present data on the composition of danmu comments and the identification and contribution of danmu subtitlers. Table 2 reveals that out of 1,490 danmu comments, 80.6% were general comments and 19.9% were danmu subtitles, with additional classifications provided. The identification and contribution of the top 10 danmu subtitlers are detailed in Table 3.

**Table 2:** Composition of danmu comments.

Danmu comments’ Category	Counts	Percentage
General comments	1,201	80.6%
- Requests for translation	- 39	2.6%
- English caption	- 22	1.5%
- Appreciation of translation	- 14	0.9%
- Other comments	- 1126	75.6%
-Danmu subtitles	296	19.9%
-- Interlingual translations	- 224	15.0%
-- Gist translations	- 67	5.5%
-- Translation revisions	- 4	0.3%
-- Translation criticism	- 1	0.1%
Totaled	1,490	100.0%

**Table 3:** Top 10 Danmu subtitlers’ identification and subtitles counts.

	Danmu subtitlers’ ID	Counts of danmu subtitles	Percentage
1	1b110846	75	26.0%
2	568b495f	30	10.4%
3	15c8b02e	17	5.9%
4	76debd77	14	4.8%
5	58494c7e	13	4.5%
6	2d10b3e9	10	3.5%
7	1ab601fd	7	2.4%
8	a8ba619	7	2.4%
9	2879eba2	6	2.1%
10	36fc72d	6	2.1%
...	...	...	...
68	...	1	0.3%
	Totaled	296	100.0%

The lack of universally accepted codes of conduct for danmu subtitling necessitates a higher degree of self-regulation. Unlike professional translations, which are typically assigned or commissioned and subject to agreements, danmu subtitling is primarily driven by personal willingness. As Borodo (2020) observed, non-professional translation activities involve translators who engage in these activities for diverse reasons, including facilitating the circulation of information, enhancing access to entertainment, engaging in creative hobbies or linguistic exercises, contributing to specific organizations’ goals, and effecting macro-scale social change through micro-scale activities.

Despite not translating the entire video, danmu subtitlers collaboratively produced several danmu subtitles. The data presented in Table 3 reveals that a total of 68 danmu subtitlers participated in the danmu subtitling efforts, collectively contributing 296 danmu subtitles. Notably, 2 danmu subtitlers stood out, having significantly contributed 75 and 30 danmu subtitles, respectively. Furthermore, 4 additional danmu subtitlers each submitted more than 10 danmu subtitles, resulting in an average of approximately 4 danmu subtitles per subtitler.

Moreover, general trust is demonstrated by the danmu comments that request and appreciate danmu subtitling. A common term frequent in danmu comments is “(wild) caption-kun” (31 out of 1,490). In contemporary online culture, this term is used for individuals who voluntarily translate original videos for viewers lacking language skills. It conveys a sense of respect, with “wild” contrasting professional organizations, “caption” denoting the danmu-enabled presentation similar to subtitles, and “kun” borrowed from the Japanese language indicating respect (Yang, 2023). Out of 1,490 danmu comments, 1,201 are general comments, including 39 requests for translation and 14 expressions of gratitude towards translators (see Table 2). For instance, danmu comment 46 inquires “有野生字幕君吗” (Any wild caption-kun?) and another danmu comment says “感谢字幕君” (Thanks to caption-kun).

The quality of work, identified as a key aspect in professional translation codes, is also affected by the principle of self-regulation in danmu subtitling. Given the involvement of multiple subtitlers, disparities in translation, terminology, and grammar may arise. These inconsistencies can undermine the overall quality of the subtitles, making them difficult for viewers to understand and potentially leading to mistrust. The case study shows that arbitrary sentence splitting and integration, punctuation usage, and other translation-related phenomena observed in danmu subtitles contribute to inconsistent quality. For example, multiple sentences are often combined into a single subtitle, resulting in a lack of synchronization with the corresponding video frame. However, danmu subtitling also allows, and even benefits from, subtitlers’ varied translation styles, including the use of bilingual subtitles and humorous tones.

### 5.2 Fine-grained analysis

By the aforementioned annotation criteria, Table 4 presents the collected results. The data indicates that ethics of representation is the most prevalent model (149, 50.3%), followed by ethics of digital technology (60, 20.3%) and ethics of communication (51, 17.2%), and then norm-based ethics (27, 9.1%). While there are only a few instances of ethics of professional commitment (5, 1.7%), a solitary case of ethics of service (1, 0.3%) is observed.

**Table 4:** Results of models of translation ethics applied in the case study.

Model of translation ethics	Counts	Percentage
Ethics of representation	149	50.3%
Ethics of service	1	0.3%
Ethics of communication	51	17.2%
Norm-based ethics	27	9.1%
Ethics of professional commitment	5	1.7%
Ethics of digital technology	60	20.3%

#### 5.2.1 Ethics of representation

The traditional focus of translation studies has long been centered on the more or less “faithful” interlingual reproduction of the source text. In this context, the ethics of representation (149, 50.3%) emerges as the most frequently referenced model. The data collected in this case study indicate that literal translations are the most direct embodiment of this representation model, covering elements such as interjections, on-screen text, common English words, and the phonetic transcription of character names. However, this does not imply that all translations are executed with meticulous precision and without any alterations.

In the case study, one notable observation is the phenomenon of repeated translations (48 out of 149), where the same original sentence is translated multiple times and often displayed within the same video frame. For example, in Example 2, the original sentence features Tamara Ecclestone, the prominent female lead of the selected documentary, expressing her feelings about her daughter. Here, three users contributed translations of the same sentence, despite differences in sentence structure and rhetorical techniques. This redundancy, a characteristic feature of danmu (Yang, 2023), can be attributed to the lack of task assignments typically found in professional translation contexts. It also highlights the self-organizing nature of danmu subtitling to some extent.

**Example 2**

Original audio: I’m happiest when she is doing what she loves to do.

Danmu subtitle 565: 我会很开心, 当她做她喜欢做的事 [I will be very happy, when she does what she loves to do].

Danmu subtitle 566: 当她做开心的事时我是最快乐的 [When she does happy things I am happiest].

Danmu subtitle 567: 她做她喜欢的事时我也很开心 [When she does what she loves I am also very happy]

#### 5.2.2 Ethics of service

The ethics of service conceptualizes danmu subtitling as a voluntary service. In Example 3, danmu subtitle 28 represents the sole instance of service ethics in the study. The primary contributor of danmu comments collectively submitted a total of 75 danmu subtitles, representing the highest quantity of danmu subtitles contributed by a single danmu subtitler in this case study. Notably, the danmu subtitle 28 marks the initial entry made by the said subtitler. Hence, danmu subtitle 28 can be viewed as a translator’s introductory message that clearly articulates the translator’s working principles with modesty.

**Example 3**

Danmu subtitle 28: 随便翻翻 大家随便看看 [Just a casual translation for everyone to take a look.]

The translator’s notes function as paratextual elements through which the translator asserts their presence, thereby relinquishing the conventional role of invisibility to communicate directly with the reader (Buendia, 2013). In Example 3, the danmu subtitler actively interacts with the audience by incorporating a “translator’s note.” This action renders the subtitler visible, similar to the role of a professional translator, guiding the audience through the dialogue.

However, it should be noted that the application of service ethics highlights a vulnerability in the method. Many elements of the model are only evident in paratexts or metatexts. Since the data primarily consists of subtitles, the study tends to classify them predominantly as representational.

#### 5.2.3 Ethics of communication

The collected data reveal that the ethics of communication, with 51 occurrences (17.2%), is the third most frequently reflected model. Emphasizing effective communication with others, the ethics of communication in danmu subtitling employ translation methods such as explication, simplification, and changes in sentence order.

In Example 4, the original audio features Tamara Ecclestone, the prominent female lead, accompanied by her spouse, Jay Rutland, as she expresses her feelings about her family, which consists of a married couple and a daughter. Three danmu subtitlers provided their communicative translations, retaining the core meanings of “family relations,” “triangle,” and “stable.”

**Example 4**

Original audio: They say that the triangle is the strongest shape known to the mankind. So we are gonna to stick to three of us.

Danmu subtitle 231: 我们的家庭关系像三角形一样稳固 [Our family relationship is as stable as a triangle.]

Danmu subtitle 232: 我们三个组成了家, 大家都说三角形是最稳定的结构 [The three of us form a family, and everyone says that triangles are the most stable structure.]

Danmu subtitle 235: 不是说三角形是最稳定的吗? 我们三人组成了这个家庭 [Isn’t it said that triangles are the most stable? The three of us formed this family.]

Mistranslations were also identified in the case study. For instance, in Example 5, the original audio discussed children’s misbehavior in a swimming pool, which caused significant disruptions during an advertisement photoshoot. However, danmu subtitle 1,297 misunderstood this and provided a mistranslation, suggesting that people can do anything in a swimming pool. Given the lack of precise entry criteria for danmu subtitling, it is reasonable to assume that individuals with limited language proficiency may volunteer for translation tasks. Substantial mistranslations can negatively impact viewership and may lead to the entire danmu subtitling being perceived as untrustworthy.

**Example 5**

Original audio: I’m sure that they’ll want to be in the pool at the wrong time, and they’ll wanna nap, eat, drink. They’ll wanna do everything at the wrong time.

Danmu subtitle 1,297: 确信在泳池里面进行活动, 人们也可以休息, 吃, 喝, 做他们想做的任何事 [Be sure to engage in activities in the swimming pool, where people can rest, eat, drink, and do anything they want.]

#### 5.2.4 Norm-based ethics

Currently, danmu subtitling lacks established and widely accepted codes of practice, resulting in its operation mainly under self-regulation. However, while certain platform-imposed limitations exist, such as the prohibition of using swear words, user-generated danmu etiquette assists danmu users in commenting. One such guideline suggests placing danmu comments at the bottom of the screen for translations if the video lacks translation, ensuring they do not cover the original subtitles (Yang, 2023).

In the case study, out of 296 danmu subtitles, 194 are set to pop up at the bottom, and 6 are at the top of the screen, comprising 67.6% of all danmu subtitles. This substantial proportion of top and bottom modes represents danmu subtitlers’ self-regulation. It is also important to note that these two modes are accessible only to users with a level 3 status or higher.

Bilingual subtitles, which concurrently display text in two distinct languages, initially found their footing in cinema productions within officially bilingual nations and international film festivals. However, they have recently regained significance as a tool to accommodate the expanding capabilities of streaming media (Gouleti, 2023). This trend is particularly evident in China’s video-on-demand platforms and cinemas, as they aim to broaden their international reach and circulation (Díaz-Cintas and Remael, 2021, p. 19). Notably, young Chinese individuals are at the forefront of this movement, influenced by the growing popularity of bilingual subtitles among amateur subtitlers who voluntarily translate foreign language videos online (Hsiao, 2014; Zhang, 2013).

As demonstrated in Example 1 above, a danmu subtitler consistently generates English captions accompanied by their corresponding Chinese translations, ultimately contributing a total of 12 pairs of danmu subtitles in the form of bilingual subtitles. The consistency in maintaining the format of bilingual subtitles demonstrates the danmu subtitlers’ intention to conform to the established norm, leveraging the unique affordances provided by danmu technology.

Speaker identification plays a pivotal role in clarifying the identity of both onscreen and offscreen speakers for clarity and the name of characters is frequently utilized to identify speakers across diverse modes and practices. In the context of film scripts, it is commonplace to precede the dialogue of a character with their name, followed by the transcribed spoken words. In closed captioning, speaker identification will be required only if necessary for comprehension (for instance, when an individual is not visible on screen but it is still evident who is speaking, speaker identification is not mandatory), with the format of the speaker’s name and a space and a colon. Similarly, speaker identification is incorporated into the display of respeaking, as emphasized by Romero-Fresco (2018, p. 100).

Danmu subtitle 904 in Example 6 effectively incorporated the translation of dialogue alongside the names of the speakers, namely Tamara (the mother) and Sophia/Fifi (the daughter), serving as speaker identifications. It is presumed that danmu subtitlers adhere to the professional translation norm of speaker identification, prioritizing fulfilling implicit duties to align with established community practices and cultural expectations.

**Example 6**

Original audio:

Sophia: Just use your muscles.

Tamara: I’m trying to use my muscles.

Danmu subtitle 904:

菲:你应该用到你的肌肉 ta:我努力用肌肉的力量了.

[Fei: You should use your muscles. ta: I tried hard to use my muscle strength.]

#### 5.2.5 Ethics of professional commitment

In the case study, a limited number of 5 danmu subtitles are identified as exemplifications of models of professional commitment. Out of these danmu subtitles, 4 pertain to translation revisions (see Example 7), while one constitutes peer criticism (see Example 8). These interventions demonstrate how danmu subtitlers operationalize their fundamental ethical aspiration to be good translators – manifested through voluntary quality assurance – while simultaneously aligning this professional ethos with personal creativity and broader social responsibility.

Example 7 illustrates self-revision as ethical self-regulation. Revision can be understood as the process of reading a translation in order to spot problematic passages, and then making or recommending any corrections or improvements that are needed to meet some standard of quality (Mossop, 2014, p. 249). Here, the predicate “sure” is corrected from “相信” (believe) to “确信” (sure) resolving a pragmatic mismatch by prioritizing evidence-based certainty over subjective belief. Crucially, this adheres to Chesterman’s ethics of professional commitment, wherein the translator voluntarily assumes accountability for terminological precision and catalyzes personal creativity as the translator innovatively negotiates pragmatic equivalence.

Example 8 demonstrates peer correction as communal commitment. Danmu subtitle 734 identifies a translation error made by danmu subtitle 732, specifically the misinterpretation of “sort of” as “soft.” Such interventions uphold collective accuracy despite contributors’ informal roles, positioning peer critique as an ethical obligation under Chesterman’s framework and it enables creative engagement. To operationalize this ethical commitment, platforms could implement peer-validation badges to incentivize community-vetted corrections.

**Example 7**

Original audio: I’m sure Fifi will have a very strong opinion on the kids that should stay and who she wants to play with.

Danmu subtitle 1,227: 我相信fifi会有自己很坚定的选择 在让哪个孩子和她一起玩这件事上 [I believe that fifi will make her own firm choice about which children to let play with her.]

Danmu subtitle 1,228: 我确信fifi会有她自己很坚定的选择 [I am sure fifi will make her own firm choice].

**Example 8**

Original audio: He hates any sort of cake.

Danmu subtitle 732: 他就是讨厌一切软软的蛋糕 [He just hates all soft cakes.]

Danmu subtitle 734: 翻译错了, 是sort of, 不是软软的 [The translation is wrong, it’s sort of, not soft.]

#### 5.2.6 Ethics of digital technology

The ethics of digital technology constitutes the second most prevalent model in danmu subtitling, accounting for 60 instances or 20.3%. Within this category, gist translation is predominant, making up 81.7% (49 out of 60), with the goal of offering generalized interpretations that are adapted to specific contexts. This can be interpreted as an omniscient perspective, that is, a third-party viewpoint. Rather than directly translating the subjects’ words, subtitlers opt for terms such as lexical bundles to denote a generalization. This method fosters a sense of detachment, enabling viewers to perceive the dialogue from a more objective position.

For instance, lexical bundles, which refer to frequently recurring sequences of words, serve as crucial structural components in both spoken and written forms of discourse (Biber and Barbieri, 2007). The collected data reveals a frequent use of specific lexical bundles including “总之就是” (This is, in a word), “这是” (this is about), “大概在聊” ((They are) approximately talking about), to summarize the presented information.

For instance, in the case of Example 9, a gist translation is employed in danmu subtitle 1,075 to concisely convey the meaning of multiple consecutive dialogues through the use of the summative lexical bundle “这里是说” (This is talking about). This approach effectively summarizes the key points of the conversation, facilitating comprehension for the viewer. It is noteworthy that the information imparted by the danmu subtitler in this instance is highly concise, condensing and omitting substantial amounts of information. Potential explanations for this could be attributed to the self-regulatory nature of danmu subtitling, which does not impose stringent subtitling standards on danmu subtitlers, to the unique characteristic of danmu comments, i.e., their temporality, or to the Bilibili platform’s expectation that danmu comments be simple and concise.

**Example 9**

Original audio:

Jay:... Tamara believes in breastfeeding. She believes quite passionately in it. I support her in anything that she wants to do. I think she’s doing the right thing. It’s the most natural thing in the world. It’s not just about food and nutrition. It’s also about comfort.

Tamara: I think that’s really important for the relationship between mother and child. I guess it’s just what was for me and Fifi, and I feel like that’s just what you have to listen to as a mom and get on with it. ...

Danmu subtitle 1,075: 这里是说她母乳喂养 [This is talking about her breastfeeding.]

In providing a general interpretation of the given information, danmu subtitlers have the discretion to choose the narrative perspective. Varying applications of narrative perspectives can yield distinct narrative effects. For instance, in Example 5, danmu subtitles 583 and 650 offer concise summaries of the female and male leads’ words regarding converting their swimming pool into an entertainment room for their daughter. Notably, there is a transition from a first-person narrative (“We”) to a third-person narrative (“They”) in the cited Example 10. This shift to a third-person narrative perspective may invoke a sense of omniscience, in contrast to the first-person narrative, which conveys a feeling of direct involvement (Morreall, 1994).

**Example 10**

Original audio: I’ve decided to turn the swimming pool at the house, which we hardly use at all, ... basically, it looks amazing, but it’s kind of a waste of space to turn into something that Sophia will definitely use on a daily basis... How excited are you? Yeah, you ready for a surprise?...

Danmu subtitle 583: 我们把不常用的泳池房改造成了菲菲一定会喜欢的娱乐房(惊喜) [We transformed the rarely used pool room into an entertainment room that Feifei will definitely like (surprise)].

Danmu subtitle 650: 他们为了女儿建了一个泳池就为了她能时而去玩 [They built a swimming pool for their daughter just so she could play there sometimes.]

According to Yin and Fun (2017, p. 132), the practice of danmu commenting on Bilibili has fostered a sense of “self-expression, participation, and empowerment” among Chinese youths, thus facilitating their engagement in the public sphere through quotidian forms of online entertainment, consumption, and interaction. In the context of this case study, it is observed that danmu subtitlers utilized humorous and engaging remarks to facilitate communication. Notably, the collected data, which reflects the communication model outlined in this study, is abundant in emotional expressions, encompassing modal particles and personal comments.

Particles are an integral part of colloquial speech, particularly in informal contexts, and modal particles are usually employed to convey modality (Chappell, 1991). For example, Example 11 uses the term “呦” (yo) to convey their intended emotions.

**Example 11**

Original audio: ... love at first sight.

Danmu subtitle 248: 呦, 还一见钟情 [Yo, it was love at first sight]

In Example 12, the dialogue originally occurred between the male lead of the documentary and his daughter, upon the father’s return from his trip and reuniting with the mother and daughter during their vacation. When the father inquired if the daughter had missed him, danmu subtitle 1,454 translated the daughter’s response of “No” and further appended interpretive comments in parentheses, reflecting the danmu subtitler’s impression regarding the daughter’s sentiment.

**Example 12**

Original audio: – Did you miss me? - No!

Danmu subtitle 1,454: 不!(好喜欢diss她爸爸 [No! (She really likes diss her father.)]

### 5.3 The emergence and resolution of ethical conflicts

When analyzed through the translation ethics framework, danmu subtitling encounters varied ethical conflicts. These conflicts manifest distinctly within the models:

#### 5.3.1 Ethics of representation

Danmu subtitling inherently challenges representation ethics due to its participatory nature. The core principle of ethics of representation -- demanding faithfully rendering source content -- is challenged, as danmu comments are inherently subjective reactions. Real-time demands further compromise fidelity, prioritizing speed over faithful representation and preventing nuanced verification.

#### 5.3.2 Ethics of service

Danmu subtitlers act for personal expression or community engagement, not professional mandates, creating ambiguity about service recipients (platforms, viewers, or creators). This lack of defined accountability frequently conflicts with creators’ rights, especially when content is reinterpreted without consent. Besides, danmu creates an ethical imbalance in labor value: platforms profit from user-generated content via engagement enhancement and data monetization, while volunteers receive only intrinsic rewards like community belonging or self-expression. This system economically benefits platforms without commensurate compensation for contributors, highlighting exploitative digital labor dynamics.

#### 5.3.3 Ethics of communication

Within the communication ethics model, danmu translation functions dually as a cross-cultural conduit and a socio-informational vehicle, extending beyond mere linguistic transfer. This dual mandate necessitates that translators serve as ethical mediators who not only convey content but critically evaluate its potential societal ramifications. Specifically, they must assess risks of intercultural misunderstandings, gauge conflict escalation potential, and mitigate communicative harm through proactive semiotic choices—balancing fidelity against social consequences in volatile digital ecosystems. Besides, the sheer volume and visual density of scrolling comments can obscure the underlying video, hindering communication and creating significant “noise.”

#### 5.3.4 Norm-based ethics

Norms governing practices are inherently culture-specific and diverge across communities and individual value systems. Traditional subtitling conventions—prioritizing accuracy, clarity, temporal synchronization, and spatial positioning—prove largely inapplicable to danmu subtitling due to its platform-contingent novelty. This emergent practice lacks established universal norms, as participatory dynamics prioritize divergent viewer expectations: some value comment density and wit for communal engagement, while others emphasize content comprehension. Such variability fundamentally contravenes the norm-based ethical principle of trust, which requires predictable behavioral consistency. Consequently, viewers cannot rely on danmu annotations for reliable comprehension assistance, unlike professional subtitles. Besides, norms emerge as contested sites where power dynamics actively dictate whose contributions gain legitimacy, inherently embedding ethical conflicts. For instance, in terms of platforms, there are norms—shaped by platform algorithms, dominant user demographics, and interface design— that privilege certain voices while marginalizing others, transforming ethical translation into a political act. This exclusion mirrors broader structures of cultural power, where assimilatory practices masquerade as “community standards.”

#### 5.3.5 Ethics of professional commitment

This translation ethics requires decisions anchored in core values—integrity, clarity, mutual understanding, and trust—yet contemporary practices demand critical alignment of professional standards, personal creativity, and social responsibility. This tripartite negotiation proves particularly salient in crowdsourced contexts like danmu subtitling, where the absence of codified professional commitment fosters fragmented ethical priorities. Participants diverge sharply: some prioritize expediency/visibility through rapid contributions; others emphasize communal engagement via humor; a minority pursues accuracy aligned with traditional ethics; while affective expression or fandom solidarity drives others. This pluralism exemplifies the central tension in digital translation: how practitioners might balance linguistic fidelity and professional accountability with creative-cultural passion within participatory ecosystems.

#### 5.3.6 Ethics of digital technology

The ethics of digital translation technology demands a critical examination of how platform affordances amplify ethical risks while introducing novel technologies. For instance, in danmu subtitling, anonymity features reduce contributor accountability, potentially compromising quality, while technological innovations -- like third-person narration perspective -- disrupt established norms despite their creative utility.

Addressing ethical conflicts in danmu subtitling necessitates adaptive regulation. This approach rejects rigid adherence to singular ethical models, instead advocating for situational awareness and deliberate choice-making by subtitlers.

Here are some application examples across ethical models. When fidelity conflicts with subjective interpretation, adaptive regulation guides subtitlers to mentally assess the source material’s vulnerability to distortion. Contributors internally interrogate service recipients: Who benefits from my comment? If cultural bridging is urgent (e.g., clarifying regional idioms), they serve viewers; if creator intent is sacred (e.g., artistic films), they self-censor reinterpretations. Danmu subtitlers assess communicative risk in real-time: Does a comment clarify cultural context (value-added) or obscure critical dialogue (noise)? When dominant practices clash with inclusivity (e.g., inside jokes excluding newcomers), adaptive regulation empowers ethical dissent—opting out or counter-commenting to uphold equitable participation. The absence of formal standards necessitates self-defined quality benchmarks. Contributors critically evaluate technological affordances, and this mindfulness turns features into conscious ethical instruments.

By centering participant agency, adaptive regulation transforms ethical conflicts from systemic constraints into deliberative practice spaces. It positions danmu subtitlers not as passive rule-followers but as reflective practitioners capable of ethical self-regulation amid fluid digital contexts.

## 6 Conclusion

Applying, modifying and extending Chesterman’s major proposition of models of translation ethics in the examination of danmu subtitling, the present study aims to explain danmu subtitling from a new theoretical perspective. Descriptive discourse analysis is performed on a case study involving an episode of an untranslated English variety show, which received 1,490 danmu comments encompassing 296 translation-related interactions.

The findings reveal that danmu subtitling is by and large a self-regulative endeavor and different ethical models play a role in elucidating danmu subtitling. Specifically, Chesterman’s framework of translation ethics can be effectively applied in danmu subtitling, revealing the significance of the five ethical models and the newly proposed model of digital technology.

Among these models, the ethics of representation, which emphasizes the preservation of truth through faithful representation, stands out as the most frequently observed, accounting for 50.3% (149 instances). Following are the newly proposed ethics of digital technology (60, 20.3%) and ethics of communication (51, 17.2%), which emphasise technological affordances and facilitating understanding and communication, respectively. Occasional instances reveal the norm-based ethics, reflecting efforts towards adhering to cultural norms and practices, attributed to the ethical virtue of trust, comprising 9.1% (27 instances). Instances of translation revisions and translation criticism, indicative of the danmu subtitlers’ commitment to excellence, are rare but illustrative of the ethics of commitment, accounting for 1.7% (5 instances). Finally, although there is only a solitary instance explicitly expressing voluntary service (ethics of service, 0.3%, 1 instance), the practice of danmu subtitling itself constitutes a form of service. Besides, this study further discusses diverse ethical conflicts arising in danmu subtitling and proposes adaptive regulation as a resolution mechanism.

This study advances scholarship by rigorously applying and extending translation ethics frameworks to danmu subtitling, thereby expanding theoretical horizons in both Media and Translation Studies. It offers novel, empirically grounded insights into the ethical dimensions of participatory audiovisual translation in the digital age, an area previously underexplored. As research on danmu subtitling gains momentum, this study establishes the ethical perspective as an essential and distinct analytical lens, providing a crucial fresh viewpoint for understanding the motivations, practices, and societal implications of this dynamic form of subtitling. The introduction and validation of the “ethics of digital technology” as an extension to Chesterman’s framework represents a key theoretical advancement. This model, responsive to the technological mediation of danmu, holds significant potential not only to enrich research in translation ethics but also to inform ethical analyses of other technologically mediated collaborative translation practices emerging in the digital landscape.

While providing valuable foundational insights, the study’s limitations stem from the use of a narrow data set. Future research should expand by encompassing diverse content genres, platforms, and user demographics. Furthermore, triangulating textual analysis with visual data, user engagement metrics, and qualitative feedback from viewers and translators will yield a more holistic and nuanced understanding of the complex ethical dynamics, inherent nature, influence, and reception of danmu subtitling. Such research will further solidify the significance of ethical frameworks for understanding digital translation phenomena.

## Data Availability

The original contributions presented in the study are included in the article/Supplementary material, further inquiries can be directed to the corresponding author/s.
